# Optimizing photovoltaic thermal (PVT) collector selection: A multi-criteria decision-making (MCDM) approach for renewable energy systems

**DOI:** 10.1016/j.heliyon.2024.e27605

**Published:** 2024-03-13

**Authors:** Sahand Hosouli, Nachiket Gaikwad, Shabahat Hasnain Qamar, Joao Gomes

**Affiliations:** a Kingston University London, UK; b MG Sustainable Engineering AB, Sweden; c University of Oldenburg, Germany; d University of Gavle, Sweden

**Keywords:** MCDM, TOPSIS, VIKOR, Target based selection, PVT selection

## Abstract

In the realm of renewable energy systems, the effective selection of Photovoltaic Thermal (PVT) collectors is important. This study delves into the intricacies of choosing optimal PVT collectors available in the market, emphasizing the utility of Multiple Criteria Decision Making (MCDM) methodologies. PVT collectors are differentiated based on various aspects such as their transparent front cover presence and the medium—liquid or air—used for heat transfer. The study methodologically identifies commercially available PVT collectors and establishes key performance indicators for comparison. Using an 11-point fuzzy scale, a weight matrix is determined. Subsequently, advanced MCDM techniques, including PROMETHEE II, TOPSIS, EDAS, and VIKOR, are employed to rank the alternatives. This research not only provides a systematic approach to PVT collector selection but also extends to a MATLAB-based tool for facilitating the process. The results underscore the value of the proposed methodologies in refining the selection of PVT collectors and their potential adaptability to other applications, such as PVT collector design.

## Highlights

• Comprehensive evaluation of ten commercially available PVT collectors, highlighting module with superior performance.
• Direct comparison of PVT modules based on their electrical efficiency, thermal efficiency, and other effective criteria.
• Application of four MCDM techniques revealed consistent module rankings, emphasizing methodological robustness.
• MATLAB-based analysis confirmed the relative importance of key performance indicators in PVT module selection.

## 1 Introduction

Photovoltaic thermal (PVT) collectors convert solar energy into both heat and electrical power. Solar cells, also known as photovoltaic cells (PV) cells, transform sunlight into electricity [1]. PVT collectors, in essence, are PV modules paired with solar thermal absorbers, enabling them to generate both heat and electricity. This dual generation of power results in higher efficiencies than if the modules worked individually as either photovoltaic or thermal units. While systems with separate solar thermal and PV modules may be more cost-effective in terms of m^2^, PVT collectors yield more energy per square meter, optimizing the energy output for available rooftop spaces [2,3]. One notable benefit of PVT collectors over traditional PV ones is their ability to maintain optimal operating temperatures, thus serving as cooling agents for PV cells, as PV cell efficiency is temperature-sensitive [[4], [5], [6]]. From the 1970s onward, extensive research has been conducted on PVT technology [4,[7], [8], [9]]. In recent times, several companies have introduced numerous PVT products, indicating a growing emphasis on this technology. The demand for PVT has witnessed a notable upswing in recent years [10,11]. PVT products, which are essentially expanded standard PV modules, dominate the market [12]. Baggenstos and his team [13] carried out a survey under the IEA-SHC Task PVT Systems, identifying PVT collector producers and suppliers in 11 nations. As per their findings [13], by the end of 2018, a total of 1.075.247 m^2^ of PVT collector space was produced. In Europe, France led the PVT market with approximately 442,504 m^2^, followed by Germany with 109,380 m^2^ [13,14].

The burgeoning PVT market makes choosing the right PVT module for specific needs a complex task with multiple influential factors. The choice of PVT module can significantly impact the success of a project [11]. This decision-making can be represented as a mathematical problem with specific variables, constraints, and objectives. Zenhäusern and colleagues [15] outlined crucial performance metrics for PVT systems in their IEA SHC Task report. These metrics are categorized into energy, environment, and economic indicators [15]. Energy indicators gauge the system's energy efficiency, environmental indicators measure its environmental impact, and economic indicators, which can profoundly affect a project's success, relate to its financial viability [15].

The focus of this study is the selection of a PVT module based on chosen performance metrics, such as electrical efficiency, zero loss efficiency, and several others, using multi-criteria decision-making techniques. Deciding optimally in any situation has always been a human challenge, and currently, modern science and technology tackle this problem [16]. Various disciplines like operations research and statistics aid in optimal decision-making [18]. One widely recognized technique is multi-criteria decision making (MCDM). MCDM has been a rapidly expanding area for at least the past two decades [16,19]. It offers a structured approach that assists in amalgamating various inputs with benefit/cost details and stakeholders' viewpoints to prioritize project alternatives [17,20].

Primarily, MCDM methods work well with quantitative criteria. However, they encounter challenges with qualitative ones [16,21]. For qualitative criteria, where a specific quantitative value isn't available, this paper adopts a ranked judgment using a fuzzy conversion scale [22]. Utilizing fuzzy set theory, criteria values can be determined linguistically, converted into corresponding fuzzy numbers, and then translated into definitive scores. Rao's 2007 research [23] presented a method built upon Cheng and Hwang's 1992 work [24].

This paper's initial segment delves into well-known MCDM techniques and their respective algorithms. Every MCDM approach is grounded in a specific mathematical rationale that recommends the optimal choice. However, this paper omits the derivation of these mathematical logics and focuses solely on the algorithmic aspect of certain methodologies. The MCDM techniques explored in this work include.

1. Preference Ranking Organization Method (PROMETHEE)-II
2. Technique for Order of Preference by Similarity to Ideal Solution (TOPSIS) method
3. Evaluation Based on Distance from Average Solution (EDAS) method
4. VIseKriterijumska Optimizacija I Kompromisno Resenje (VIKOR)

In the subsequent sections, PVT modules available in the market are assessed based on key performance indicators sourced from manufacturers' datasheets. This paper utilizes an eleven-point fuzzy scale for evaluating alternative criteria. The method proposed by S.-J. Chen and C.-L. Hwang [23] transforms fuzzy values (linguistic evaluations) into definite scores, and the results derived from various MCDM techniques are showcased.

Outranking Methods (OMs), a subset of MCDM techniques, construct a preference hierarchy, commonly referred to as an outranking relation, between alternatives evaluated across multiple attributes [25]. This relation in most OMs is derived through a series of pairwise alternative comparisons. Outranking techniques originated in France during the late 1960s due to challenges faced when employing the value function approach for real-world issues [26]. The Preference Ranking Organization Method (PROMETHEE)-II, highlighted in this document, serves as an OM exemplar [27].

Embarking on MCDM problems primarily involves formulating a decision matrix. Decisions are driven by this matrix, which facilitates the evaluation and ranking of potential choices. This matrix is often the starting point for addressing decision-making challenges. Stakeholders initially draft a weighted set of criteria to juxtapose every option against. Although all methods initiate with this assessment matrix, their distinctions arise based on additional data requisites. The criteria can be bifurcated into two categories: beneficial criteria, which aim for maximization or higher values, and non-beneficial or cost criteria, which strive for minimization or lower values. The decision matrix can be denoted as [x_ij_]_m×n_, with "n" representing alternatives and "m" indicating criteria.

(1) $${\left[\;x_{ij}\right]}_{m\times n}=\left|\begin{array}{ccc} X_{11} & X_{12\;}\;.\;.\;.\; & X_{1m}\\ X_{21} & X_{22}\;.\;.\;\text{.} & X_{2m}\\ {\begin{array}{c} .\\ .\\ .\\ X \end{array}}_{n1} & \;{\begin{array}{c} .\\ .\\ .\\ X \end{array}}_{n\;.\;.\;.} & {\begin{array}{c} .\\ .\\ .\\ X \end{array}}_{nm} \end{array}\right|$$

Here, [x_ij_]_m×n_ signifies the performance value of the i_th_ alternative against the j_th_ criterion (i ∈ 1, 2, …, m) and (j ∈ 1, 2, …, n).

Given these distinctions and the intricate task of pinpointing PVT collectors suited for particular applications, this study's objective is to holistically comprehend the selection dynamics. Central aims include the systematic identification of PVT collector options, the development of a comprehensive criteria and performance indicator set, and the application of advanced MCDM techniques like PROMETHEE II, TOPSIS, EDAS, and VIKOR for a transparent and insightful ranking of these options. This research thereby contributes to crafting a structured decision-making blueprint for PVT collector selection, which is versatile for multiple renewable energy contexts. Moreover, it underscores the feasibility of the suggested methodologies in navigating renewable energy system design and operational challenges.

## 2 Preference Ranking Organization Method (PROMETHEE)-II

Brans [30] first presented the PROMETHEE I and PROMETHEE II techniques. The PROMETHEE II approach yields a full ranking, while PROMETHEE I only gives a partial sequence of the potential choices. The PROMETHEE methodologies have been effectively used across diverse sectors such as banking, industrial location, manpower planning, water resources, investments, medicine, chemistry, health care, tourism, ethics in operations research (OR), and dynamic management [[32], [33], [34], [35]]. The system's mathematical characteristics and straightforwardness are largely behind its popularity. For real-world scenarios, Brans et al. [35] recommended employing both PROMETHEE I and PROMETHEE II. However, the PROMETHEE II approach is usually more effective in establishing a full ranking of the given options [36,37].

**Fig. 1:**
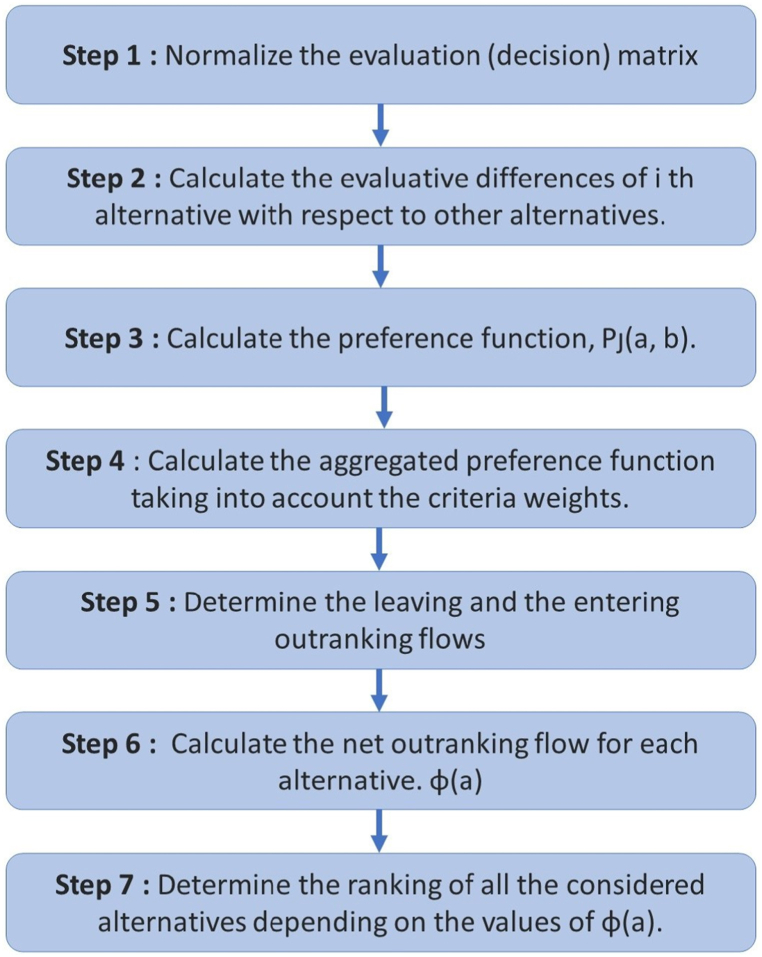
Flowchart showing the main steps in PROMETHEE II.

The procedure for the PROMETHEE II technique is outlined below and Fig. 1 [29,31].

Step 1 Utilize equation (2) to normalize the evaluation (decision) matrix,

(2) $$\frac{\left[x_{ij}\;-\min\left(x_{ij}\right)\right]}{\left[\max\left(x_{ij}\right)-\min\left(x_{ij}\right)\right]}\left(i=1,2...m;\;j=1,2,...n\right)$$

where *x*_*ij*_ is the performance measure of *i*th alternative with respect to *j*th criterion and *R*_*ij*_ is the normalized value of *x*_*ij*_. For non-beneficial criteria, the above equation can be rewritten by equation (3):

(3) $$R_{ij}=\frac{\left[\max\left(x_{ij}\right)\;-x_{ij}\right]}{\left[\max\left(x_{ij}\right)\;-\;\min\left(x_{ij}\right)\right]}$$

where *n* is the number of evaluation criteria, min *x*_*ij*_ and max *x*_*ij*_ are the minimum and maximum values of *x*_*ij*_, respectively. This normalization procedure is required to make the criteria values dimensionless and comparable. After normalization, all the criteria values should lie between 0 and 1.

Step 2 Determine the evaluative variances between the *i*th alternative and its counterparts. This entails figuring out the differences in criteria values among various options on a pairwise basis.

Step 3 Compute the preference function, *P*_*j*_*(a,b)*. While there exist six generalized preference functions as mentioned in Ref. [22], they typically need the establishment of certain preferential parameters, like indifference and preference thresholds. Yet, in real-time scenarios, pinpointing a specific form of preference for the decision-maker to ascertain the values of these parameters can be challenging. To circumvent this issue, the preference function outlined in equation (4) is used in this context.

(4) *P*_*j*_(*a*, *b*) = 0 if *R*_*aj*_ ≤ *R*_*bj*_

(5) *P*_*j*_(*a*, *b*) = *R*_*aj*_ − *R*_*bj*_ if *R*_*aj*_ *>* *R*_*bj*_

Step 4 Calculate the aggregated preference function taking into account the criteria weights. Aggregated preference function, *π (a, b)*

(6) $$\pi \left(a,b\right)=\frac{\left[\sum_{j=1}^{n}w_{j}P_{j}\left(a,b\right)\right]}{\sum_{j=1}^{n}w_{j}}$$

Step 5 Determine the leaving and the entering outranking flows by equation (7), Leaving (positive) flow for *a*th alternative,

(7) $$\varphi^{+}\left(a\right)=\frac{1}{m-1}\sum_{b=1}^{m}\pi \left(a,b\right)\;\left(a\neq b\right)$$

Step 6 Calculate the net outranking flow for each alternative.

(8) $$\varphi {\left(a\right)=\varphi }^{+}\left(a\right)-\;\varphi^{-}\left(a\right)$$

where *φ(a)* is the net outranking flow value for alternative *a*.

Step 7 Determine the ranking of all the considered alternatives depending on the values of *φ(a)*. The higher the value of *φ(a)*, the better is the alternative.

## 3 Technique for Order of Preference by similarity to ideal solution (TOPSIS) method

**Fig. 2:**
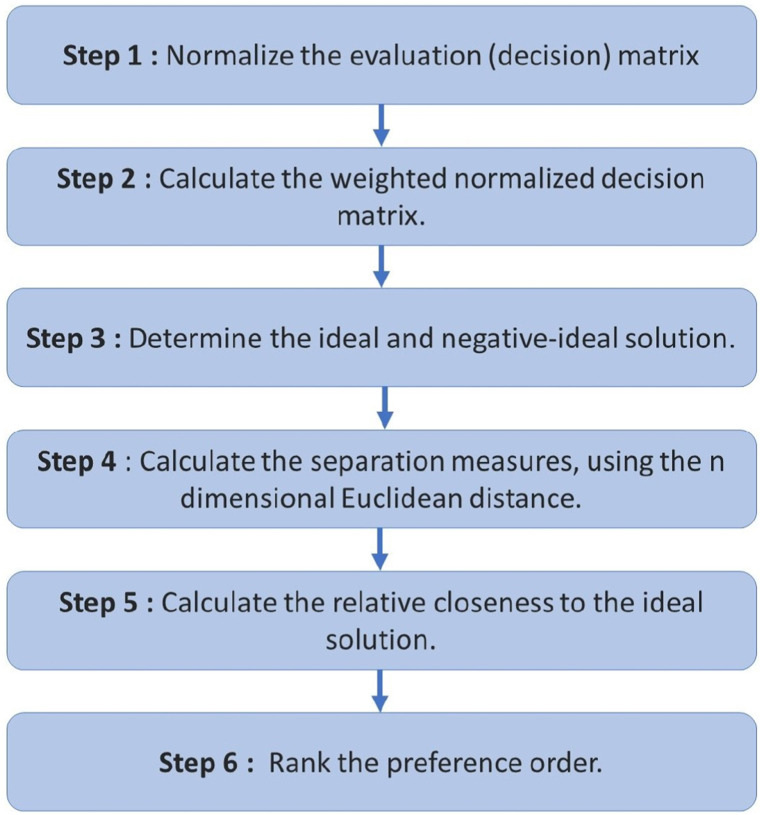
Flowchart showing the main steps in TOPSIS.

The Technique for Order of Preference by Similarity to Ideal Solution (TOPSIS) was initially introduced by Ching-Lai Hwang and Yoon in 1981 [38]. It was further refined by Yoon in 1987 [39] and later by Hwang, Lai, and Liu in 1993 [40]. Central to TOPSIS is the principle that the chosen option should be nearest to the positive ideal solution (PIS) and at the greatest distance from the negative ideal solution (NIS) [41]. This method of compensatory aggregation evaluates a range of options by setting weights for each criterion, standardizing scores for each one, and gauging the geometric distance between each option and the ideal one, defined as the option with the top score for every criterion. TOPSIS posits that criteria are either monotonically increasing or decreasing [42]. In contexts with multiple criteria, normalization is often essential because the parameters or criteria usually have different dimensions [40]. The procedure for the TOPSIS approach is detailed as follows (see Fig. 2) [39].

Step 1 Normalize the evaluation (decision) matrix.

Step 2 Calculate the weighted normalized decision matrix. The weighted normalized value *v*_*ij*_ is calculated as *v*_*ij*_ *=* *w*_*i*_*r*_*ij*_, *j* *=* 1, …, *J*; *i* *=* 1, …, *n*, where *w*_*i*_ is the weight of the *i*th attribute or criterion, and $\Sigma_{i=1}^{n}wi=1$.

Step 3 Determine the deal and negative-ideal solution.

(9) $$A^{*}=\left\{v_{1}^{*},.\;.\;.\;.,v_{n}^{*}\right\}=\left\{\left(\max\;v_{ij}|i\in I^{\prime }\right),\left(\min\;v_{ij}|i\in I^{\prime \prime }\right)\right\}$$

(10) $$A^{-}={\{v}_{1}^{-},.\;.\;.\;.,v_{n}^{-}\}=\{\left(\max\;v_{ij}|i\in I^{\prime }\right),\left(\min\;v_{ij}|i\in I^{\prime \prime }\;\right)\}$$

where *I′* is associated with benefit criteria, and *I″* is associated with cost criteria.

Step 4 Calculate the separation measures, using the n dimensional Euclidean distance. The separation of each alternative from the ideal solution is given by equation (11)

(11) $${\;D}_{\begin{array}{c} {}*\\ j \end{array}}=\sqrt{\sum_{i=1}^{n}{\left(v_{ij}-v_{\begin{array}{c} {}*\\ i \end{array}}\right)}^{2}},j=1,\ldots ,J$$

Similarly, the separation from the negative ideal solution is by equation (12)

(12) $$D_{\begin{array}{c} -\\ j \end{array}}=\sqrt{\sum_{i=1}^{n}{\left(v_{ij}-v_{\begin{array}{c} -\\ i \end{array}}\right)}^{2}},j=1,\ldots ,J$$

Step 5 Calculate the relative closeness to the ideal solution. The relative closeness of the alternative *a*_*j*_ with respect to A*∗* is defined as

(13) $$C_{j}^{*}=D_{j}^{-}/\;D_{j}^{*}+D_{j}^{-},j=1,.\;.\;.,J$$

Step 6 Rank the preference order.

## 4 Evaluation based on distance from average solution (EDAS) method [43]

The method called evaluation based on distance from average solution (EDAS) was proposed by Keshavarz Ghorabaee et al. [43] specifically for inventory ABC categorization. The evidence suggests that the EDAS approach boasts notable efficiency and requires less computational effort than other ABC categorization techniques. In addition, when pitted against several prevalent methods, the prowess of the EDAS technique as an MCDM method was evident. The technique assesses alternatives based on how each deviates from the mean solution concerning each criterion [41].

**Fig. 3:**
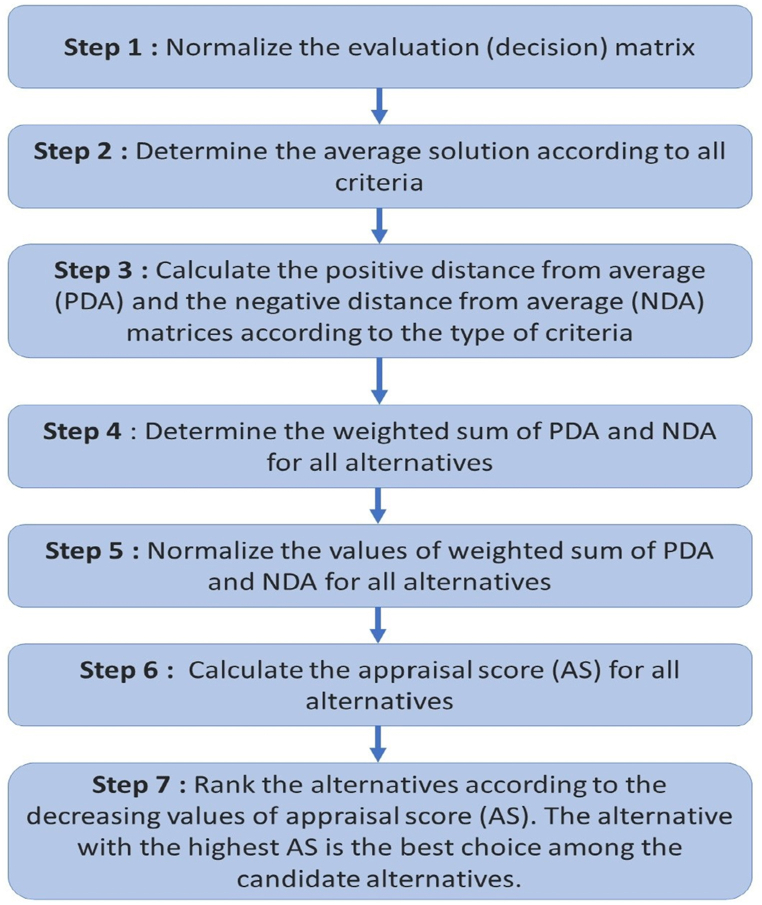
Flowchart showing the main steps in EDAS.

The steps for EDAS method are listed as follows (see Fig. 3).

Step 1 Normalize the evaluation (decision) matrix.

Step 2 Determine the average solution according to all criteria, as given by equation (14).

(14) $$AV={\left[{AV}_{j}\right]}_{\;1\times m^{\prime }}$$

where,

(15) $${AV}_{j}=\frac{n_{i=1}\;x_{ij}}{n}$$

Step 3 Calculate the positive distance from average (PDA) and the negative distance from average (NDA) matrices according to the type of criteria (benefit and cost), is given by equation (16).

$$PDA={{[PDA}_{ij}]}_{n\times m}$$

(16) $$NDA={{[NDA}_{ij}]}_{n\times m}$$

if *j*th criterion is beneficial,

$${PDA}_{ij}=\frac{\max\;\left(0,\left(X_{ij}-{AV}_{j}\right)\right)}{{AV}_{j}}$$

(17) $${NDA}_{ij}=\frac{\max\;\left(0,\left({AV}_{j}-X_{ij}\right)\right)}{{AV}_{j}}$$

and if *j*th criterion is non-beneficial,

$${PDA}_{ij}=\frac{\max\;\left(0,\left({AV}_{j}-X_{ij}\right)\right)}{{AV}_{j}}$$

(18) $${NDA}_{ij}=\frac{\max\;\left(0,\left(X_{ij}-{AV}_{j}\right)\right)}{{AV}_{j}}$$

where *PDA*_*ij*_ and *NDA*_*ij*_ denote the positive and negative distance of *i*th alternative from average solution in terms of *j*th criterion, respectively.

Step 4 Determine the weighted sum of PDA and NDA for all alternatives, given by equation (16),

$${SP}_{i}=\sum_{j=1}^{m}w_{j}{PDA}_{ij}$$

(19) $${SN}_{i}=\sum_{j=1}^{m}w_{j}{NDA}_{ij}$$

where *w*_*j*_ is the weight of *j*th criterion.

Step 5 Normalize the values of SP and SN for all alternatives, shown by equation (20),

$${NSP}_{i}=\frac{SP}{\max_{i}{SP}_{i)}}$$

(20) $$N{SN}_{i}=1-\frac{SN}{\max_{i}{SN}_{i)}}$$

Step 6 Calculate the appraisal score (AS) for all alternatives, shown by equation (21),

(21) $${AS}_{i}=\frac{1}{2}\left({NSP}_{i}+{NSN}_{i}\right)$$

where 0 ⩽ *AS*_*i*_ ⩽ 1.

Step 7 Rank the alternatives according to the decreasing values of appraisal score (*AS*). The alternative with the highest AS is the best choice among the candidate alternatives. We can classify the alternatives with respect to this ranking.

## 5 VIseKriterijumska Optimizacija I kompromisno Re- senje (VIKOR)

**Fig. 4:**
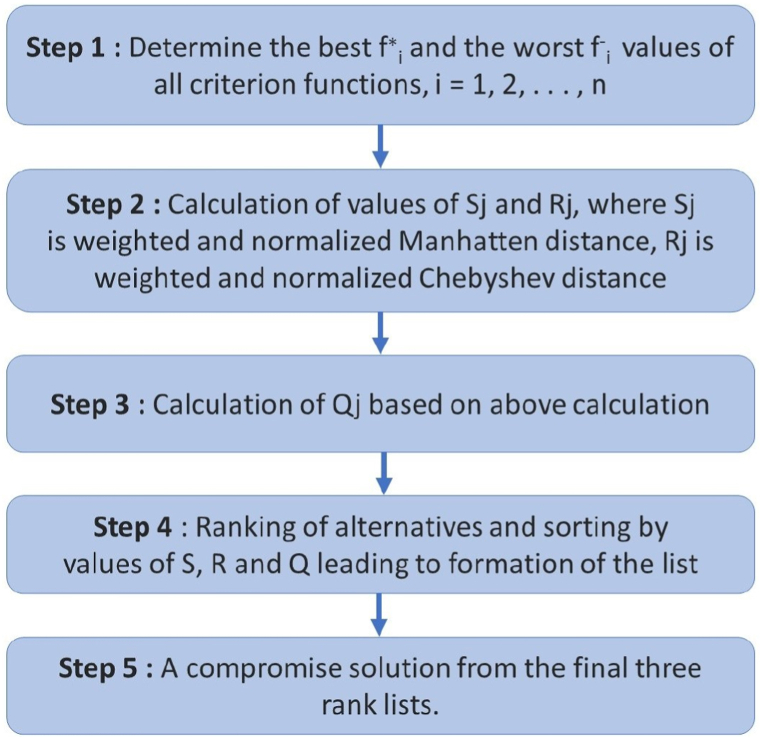
Flowchart showing the main steps in VIKOR.

The VIKOR technique was formulated for the multicriteria optimization of intricate systems [41] (see Fig. 4). It ascertains the compromise rank-order, the compromise answer, and the intervals of weight stability for maintaining the preference of the compromise outcome derived from the original (specified) weights. This technique emphasizes ordering and picking from a group of options when faced with contradictory criteria. It brings forth a multicriteria ranking indicator grounded on a specific measure of "proximity" to the "optimal" outcome [41,44]. The procedures for the EDAS method are outlined in Fig. 4 [44].

Step 1 Determine the best *f*_*i*_*∗* and the worst *f*_*i*_^*−*^ values of all criterion functions, *i =* 1, 2, …, *n*. If the *i*th function represents a benefit then: *f*_*i*_∗ *=* max_*j*_ *f*_*ij*_, *f*_*i*_^−^ *=* min_*j*_ *f*_*ij*_.

Step 2 Compute the values *S*_*j*_ and *R*_*j*_, *j* *=* 1, 2, …, *J*, by the relations given by equations (22), (23)

(22) $$S_{j}=\sum_{i=1}^{R}w_{i}\left(f_{i}^{*}-f_{i}\right)/\left(f_{i}^{*}-f_{i}\right)$$

(23) $$R_{j}=\max\;\left[\frac{w_{i}\left(f_{i}^{*}-f_{ij}\right)}{f_{i}^{*}-f_{i}^{-}}\right]$$

where *w*_*i*_ are the weights of criteria, expressing their relative importance.

Step 3 Compute the values *Q*_*j*_, *j =* 1, 2, …, *J*, by the relations given by equations (24) and (25),

(24) $$Q_{j}=v\left(S_{j}‐S^{*}\right)\;/\;\left(S^{‐}‐S^{*}\right)+\left(1‐v\right)\left(R_{j}‐R^{*}\right)/\left(R^{‐}‐R^{*}\right)$$

Where, S∗ = min_*j*_ *S*_*j*_*, S*^*−*^ = max_*j*_ *S*_*j*_*, R*∗ = min_*j*_ *R*_*j*_*, R*^*−*^ = max_*j*_ *R*_*j*_ and *v* is introduced as weight of the strategy of "the majority of criteria" (or "the maximum group utility"), here *v* = 0.5.

Step 4 Rank the alternatives, sorting by the values *S*, *R* and *Q*, in decreasing order. The results are three ranking lists.

Step 5 Propose as a compromise solution the alternative (*a′*) which is ranked the best by the measure Q (minimum) if the following two conditions are satisfied: C1 Acceptable advantage:

(26) $$Q\left(a^{\prime \prime }\right)-Q\left(a^{\prime }\right)\geq DQ$$

where *a′′* is the alternative with second position in the ranking list by *Q*; *DQ* = 1/(*J*- 1); *J* is the number of alternatives C2. Acceptable stability in decision making: Alternative a′ must also be the best ranked by *S* or/and *R*. This compromise solution is stable within a decision-making process, which could be voting by majority rule (when *v* > 0.5 is needed), or by consensus *v* ≈ 0.5, or with veto (*v* < 0.5). Here, *v* is the weight of the criteria (or the maximum group utility). If one of the conditions is not satisfied, then a set of compromise solutions is proposed, which consists of: Alternatives *a′* and *a′′* if only condition C2 is not satisfied, or Alternatives a′, a′′, …, a^(M)^ if condition Cl is not satisfied; and a^(M)^ is determined by the relation ${Q(a}^{\left(M\right)})‐Q\left(a^{\prime }\right)\;<\;\text{DQ}$ for maximum M (the positions of these alternatives are in closeness).

## 6 Application of MCDM for the selection of a PVT collector

### 6.1 Introduction and problem statement

As discussed in section 1, PVT collectors are a significant leap in the pursuit of sustainable energy sources. They produce electricity as well as heat from incident solar energy, offering higher surface yields compared to traditional PV collectors. Their dual energy harnessing capacity positions them as a promising technology in sustainable energy generation.

However, a prevailing challenge emerges: the selection of the optimum PVT collectors from an array of options in the market. PVT collectors come in a variety of forms, presenting diverse designs and functionalities. For the purpose of reducing heat losses, a distinction is made between collectors with and without a transparent front cover [3]. Additionally, they can differ based on the heat transfer medium employed, be it a liquid or air. The market overview carried out by Zenhaeusern et al. in Ref. [12] highlights that a majority of the products (38) are uncovered liquid-cooled PVT collectors. Further complications arise when considering the nature of heat as a form of energy. Being constrained by the second law of thermodynamics, the temperature at which heat is supplied critically impacts its exergy, which indicates the maximum useful work extractable. This exergy consideration compounds the intricacies of selecting the appropriate PVT collector for a specific application.

The primary challenge this research seeks to address lies in these complexities. The aim is to establish a systematic and data-driven approach to evaluate and select PVT collectors that best align with specific application requirements. Given the diverse nature of available PVT collectors and the multifaceted considerations of energy systems, there's a critical need for a structured selection methodology.

Our approach delves into the following objectives: Identifying commercially available PVT collectors in the market, defining key performance criteria grounded on established standards and guidelines, and applying advanced MCDM methodologies to provide a holistic evaluation of the available collectors. By integrating these objectives, we aim to offer a comprehensive solution to the challenges faced in PVT collector selection. Our methodology ensures a more informed decision-making process, allowing for efficient and effective deployment of renewable energy systems. This research emphasizes the complexities surrounding PVT collector selection and offers a novel approach that integrates market surveying, performance evaluation, and decision-making methodologies. We anticipate that this integrated method will substantially benefit future activities in the area of renewable energy, particularly in the design and deployment of PVT collectors.

### 6.2 Methodology

**Fig. 5:**
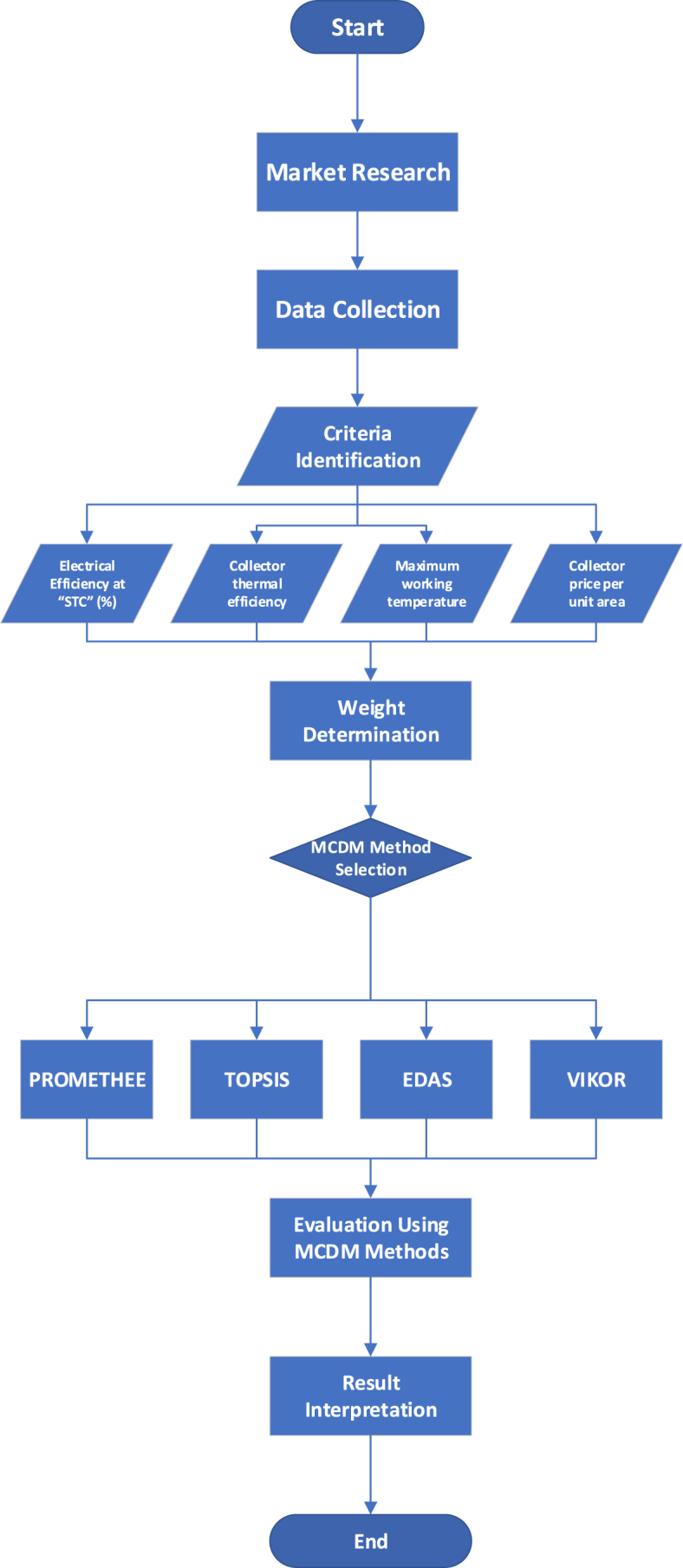
Flowchart illustrating the step-by-step methodology for PVT collector selection using multiple MCDM methods.

The outlined approach includes three primary steps: (1) Recognizing the existing market alternatives, (2) Choosing the essential criteria or performance indicators pertaining to PVT systems, (3) Formulating the weight matrix with an 11-point fuzzy scale, and (4) Leveraging PROMETHEE II, TOPSIS, EDAS, and VIKOR techniques to rank the alternatives (Fig. 5). The use of the MATLAB computational tool is noteworthy for its advanced analytical strength, ensuring an in-depth and precise evaluation of PVT collectors based on the defined criteria.

In the MCDM domain, a variety of strategies can be utilized to assess, rank, and opt for the best alternatives based on several criteria. For this research, the four prevalent MCDM methods chosen were PROMETHEE, TOPSIS, EDAS, and VIKOR. The choice of these MCDM techniques is rooted in their unique merits and relevance to different dimensions of decision-making.

PROMETHEE is distinguished by its ability to navigate conflicting criteria. Using a pairwise comparison method, it provides a more in-depth insight into the performance of each alternative relative to the others. Moreover, PROMETHEE's outranking technique sets it apart from distance-based methods, offering a distinct viewpoint on alternative rankings [[32], [33], [34], [35]]. PROMETHEE II is an efficient outranking method that has been successfully applied in various MCDM problems. Its strength lies in its ability to handle a large number of alternatives and criteria. However, it requires a priori knowledge of the criteria weights, which can be a stringent assumption. Moreover, the number of preference indices to be evaluated grows enormously with an increase in the number of alternatives [45]. TOPSIS, a pillar in MCDM, gauges alternatives based on their proximity to a perfect solution and their distance from a least ideal scenario. This method elucidates how close each PVT collector is to being the top pick [[39], [40], [41]]. TOPSIS, on the other hand, is based on the concept that the chosen alternative should have the shortest geometric distance from the positive ideal solution and the longest geometric distance from the negative ideal solution. It is praised for its simplicity, rationality, comprehensibility, good computational efficiency, and ability to measure the relative performance for each alternative in a simple mathematical form. However, it has been criticized for its potential issues with criteria and alternatives interdependence [[46], [47], [48]]. EDAS is another MCDM method that has been noted for its applicability and fewer number of calculations compared to other methods [49]. EDAS sorts alternatives based on the distance from an average solution, beneficial in scenarios where very high or very low solutions might not be the most revealing, particularly in markets with comparable product performances [41]. EDAS is another MCDM method that has been noted for its applicability and fewer number of calculations compared to other methods [49]. VIKOR, crafted for decisions with clashing criteria, places emphasis on both majority and minority factors. Merging the advantages of compromise solutions, it ranks aiming for a midpoint between the "nearest-to-ideal" and the "farthest-from-negative-ideal" solutions, leading to a well-rounded assessment of the PVT collectors [41,44]. VIKOR was developed to solve decision problems with conflicting and no commensurable criteria. It assumes that compromise is acceptable for conflict resolution and that the decision maker wants a solution that is the closest to the ideal. It has been praised for its ability to provide a ranking procedure for positive attributes and negative attributes. However, it has been criticized for containing too many pairwise comparisons and leading to inconsistencies between criteria and classification [50].

In a comparative study, it was found that the rankings obtained by these methods can vary depending on the specific decision-making context. Therefore, the selection of the most appropriate method depends on the specific requirements and constraints of the decision problem at hand. The selection of these methods was motivated by their synergistic capabilities. Each provides a unique perspective for evaluating PVT collectors, ensuring a solid, all-encompassing, and diverse analysis. By harnessing this collection of methods, the intention is to address the various complexities present in the selection phase and deliver a more consistent and trustworthy suggestion.

#### 6.2.1 Identification of the available alternatives in the market

Based on the market survey conducted by the authors, the PVT collectors available commercially were catalogued. Using datasheets for each module provided by their respective manufacturers, these modules have been referenced using generic names to safeguard the interests of the manufacturers and prevent any potential conflicts of interest.

#### 6.2.2 Identification of the criteria or key performance indicators related to PVT systems

The primary performance indicators associated with individual PVT modules were identified by reviewing the PVT modules' certification standards and the guidance given in the references. The following criteria were taken into account [[12], [13], [14]].

i. Electrical Efficiency at "STC" (%): This measure denotes the electrical efficiency of the photovoltaic component within the PVT collector under standard test conditions. This directly characterizes the electrical output anticipated, typically the main goal.
ii. Collector Thermal Efficiency (η): This efficiency represents the proportion of incoming radiation that the working fluid in a solar thermal collector absorbs. The formula for determining collector efficiency is:

(27) η=η0−a1*(Tm−Ta)G−a2(Tm−Ta)2G

Where, *η* = Collector thermal efficiency, *η*_*0*_ = Zero loss efficiency, *T*_*m*_ = Mean fluid temperature, *T*_*a*_ = Ambient temperature, *a*_*1*_ = First order heat loss coefficient, *a*_*2*_ = Second order heat loss coefficient, the expression ($T_{m}-T_{a}$/G) is referred to as the collector performance coefficient. In the theoretical analysis, the performance of the system is predicted by solving the mathematical equations by varying the values of the following parameters: ambient air temperature (*T*_*a*_), collector's mean fluid temperature (*T*_*m*_) and global solar radiation (G) denotes the measurement of the temperature difference between the collector and its surroundings relative to the solar radiation.

It can be observed that the governing equation for collector thermal efficiency is a complex one, involving a lot of parameters. Also, it is a function of incident radiation and ambient temperature, which makes it difficult to use as a comparison parameter. Hence, *η*_*0*_, *a*_*1*_ and *a*_*2*_ are used as criteria to compare selected PVT modules.

From the equation it is clear that while *η*_*0*_ is a beneficial criterion, *a*_*1*_ and *a*_*2*_ are non-beneficial criteria.

iii. Maximum working temperature: This is an important parameter because this greatly influences the output temperature desired. Higher operating temperatures facilitate higher output temperatures thus reducing the specific mass flow of absorbing fluid.
iv. Collector price per unit area: Collector price is an important criterion. This greatly affects the economic feasibility of the project and is one of the major challenges in implementation of PVT on a commercial scale. The prices of the selected collectors were obtained at the time of publishing this paper and published in the decision matrix in a normalized form. The prices have been specifically mentioned in normalized form as they are greatly affected by time, inflation, location and production technologies. This avoids ambiguity and provides price as a relative ratio rather than an absolute value. The available alternatives, named as M1, M2, M3 … M10 and the decision making criteria are summarized in Table 1.

**Table 1:** PVT modules and their criteria values.

PVT Modules	Electrical Efficiency at “STC” (%)	Zero Loss Efficiency (η_0_)	*First Order Coefficient (a*_*1*_*) [W/*$\left(m^{2}K\right)]$	Second Order *Coefficient (a*_*2*_*) [W/(*$m^{2}K^{2})]$	Maximum Working Temperature [degC]	Price (relative)
M1	17.8	0.700	5.980	0.0001	126.00	7.44
M2	12.0	0.470	3.780	0.014	180.00	8.27
M3	17.2	0.510	11.400	0.0001	74.70	16.46
M4	15.1	0.475	8.370	0.586	101.00	9.58
M5	12.9	0.486	4.028	0.067	134.00	9.67
M6	19.0	0.534	8.370	0.586	93.00	9.13
M7	20.4	0.680	10.040	0.0001	83.00	8.52
M8	17.1	0.487	5.770	0.009	95.00	10.08
M9	15.3	0.550	6.300	0.080	85.00	12.02
M10	18.4	0.470	9.500	0.0001	85.00	8.83

In the provided Table 1, module M7 exhibits the highest electrical efficiency among the alternatives. However, the decision-making process in MCDM considers a range of criteria to ensure a holistic evaluation. These criteria include not only electrical efficiency but also collector thermal efficiency, maximum working temperature, and collector price per unit area.

#### 6.2.3 Conversion of linguistic terms into crisp scores (seven-point scale)

For specific MCDM techniques, there's a need to transform qualitative data into quantifiable metrics. This article introduces a ranking value judgment using a fuzzy conversion scale, particularly for criteria that are qualitative in nature, meaning when there's no available quantitative value. Initially, these criteria are represented as linguistic expressions. They are then converted into corresponding fuzzy numbers and ultimately transformed into clear-cut scores through the application of fuzzy set theory. Utilizing the detailed numerical approximation technique, linguistic expressions are universally transformed into their respective fuzzy numbers.

**Fig. 6:**
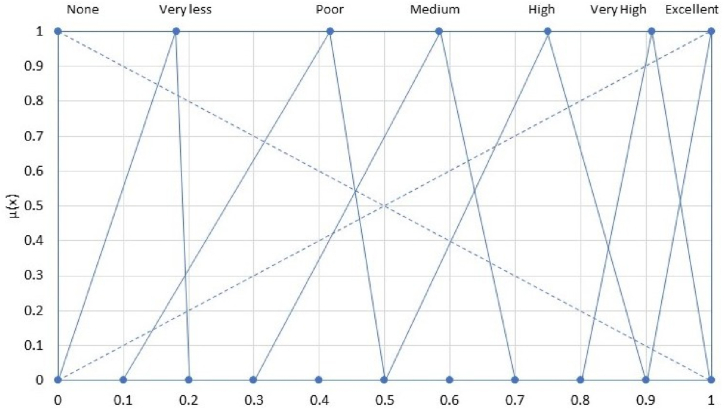
Fuzzy number conversion from linguistic terms [28].

Table 2 displays the selection criteria based on a qualitative scale via fuzzy logic, which aligns with the fuzzy conversion scale depicted in Fig. 6, serving as a means for value assignment.

**Table 2:** Conversion of linguistic terms to fuzzy numbers and corresponding crisp scores.

Linguistic Term	Fuzzy Number	Crisp Score
None (M1)	(0,0,0)	0
Very low (M2)	(0,0.1,0.2)	0.1364
Poor (M3)	(0.1,0.3,0.5)	0.3333
Medium (M4)	(0.3,0.5,0.7)	0.5
High (M5)	(0.5,0.7,0.9)	0.6667
Very high (M6)	(0.8,0.9,1)	0.8636
Excellent (M7)	(1,1,1)	1

#### 6.2.4 Creation of a decision matrix and its normalization

The data of available alternatives and their corresponding decision variables as summarized in Table 1 is used to obtain a decision matrix [*x*_*ij*_] as given by equation (28).

(28) $$\left[x_{ij}\right]=\left[\begin{array}{cccc} 17.8 & 0.700 & 5.98 & \begin{array}{ccc} 0.001 & 126.0 & 7.44 \end{array}\\ 12.0 & 0.470 & 3.78 & \begin{array}{ccc} 0.014 & 180.0 & 8.270 \end{array}\\ 17.2 & 0.510 & 11.4 & \begin{array}{ccc} 0.001 & 74.70 & 16.46 \end{array}\\ \begin{array}{c} 15.1\\ 12.9\\ 19.0\\ 20.4\\ 17.1\\ 15.3\\ 18.4 \end{array} & \begin{array}{c} 0.475\\ 0.486\\ 0.534\\ 0.680\\ 0.487\\ 0.550\\ 0.470 \end{array} & \begin{array}{c} 8.37\\ 4.03\\ 8.37\\ 10.04\\ 5.77\\ 6.30\\ 9.50 \end{array} & \begin{array}{ccc} \begin{array}{c} 0.586\\ 0.067\\ 0.586\\ 0.001\\ 0.009\\ 0.080\\ 0.001 \end{array} & \begin{array}{c} 101.0\\ 134.0\\ 93.00\\ 83.00\\ 95.00\\ 85.00\\ 85.00 \end{array} & \begin{array}{c} 9.58\\ 9.67\\ 9.13\\ 8.52\\ 10.08\\ 12.02\\ 8.83 \end{array} \end{array} \end{array}\right]$$

The decision matrix is then normalized as given by equations (2), (3).

(29) $$\left[R_{ij}\right]=\left[\begin{array}{cccccc} 0.69 & 1 & 0.711 & 1 & 0.487 & 0\\ 0 & 0 & 1 & 0.978 & 1 & 0.092\\ 0.619 & 0.174 & 0 & 1 & 0 & 1\\ 0.369 & 0.022 & 0.398 & 0 & 0.25 & 0.237\\ 0.107 & 0.07 & 0.967 & 0.887 & 0.563 & 0.247\\ 0.833 & 0.278 & 0.398 & 0 & 0.174 & 0.187\\ 1 & 0.913 & 0.178 & 1 & 0.079 & 0.12\\ 0.607 & 0.074 & 0.739 & 0.986 & 0.193 & 0.293\\ 0.393 & 0.348 & 0.669 & 0.865 & 0.098 & 0.508\\ 0.762 & 0 & 0.249 & 1 & 0.098 & 0.154 \end{array}\right]$$

The weight vector for decision criteria is obtained after conversion from linguistic variables is given as equation (30).

(30) *w*_*i*_ = [0.166, 0.212, 0.190, 0.214, 0.036, 0.182]

The weight factor matrix (*w*_*i*_) plays a crucial role in multicriteria decision-making, as it assigns importance to various criteria influencing the overall assessment. In this context, the weight vector [0.166, 0.212, 0.190, 0.214, 0.036, 0.182] indicates the relative significance of the criteria for selecting and evaluating PVT panels. The parameter with the highest weight, 0.214, corresponds to the "Second Order Coefficient (*a*_*2*_)," suggesting that this factor has the most substantial impact on the decision-making process in selecting PVT panel for elevated temperature application. This implies that variations in the second-order coefficient have a more pronounced effect on the overall performance of solar panels compared to other parameters at elevated temperature. Following closely is the parameter associated with "Zero Loss Efficiency" which carries a weight of 0.212, indicating its considerable influence on decision-making. The "First Order Coefficient (*a*_*1*_)" parameter, with a weight of 0.190, also holds significant importance, though slightly less than the previous two. The "Electrical Efficiency" parameter follows with a weight of 0.166, suggesting its moderate impact. The "Price (relative)" parameter, with a weight of 0.182, holds importance but to a lesser degree compared to the others. It's important to note that the statement regarding the importance of the price parameter, is specific to the current context and criteria established for the evaluation of this study. In alternative scenarios where economic considerations hold greater weight or are the primary focus, the importance of the price parameter might be elevated in the ranking. Economic-centric evaluations may result in assigning a higher weight to the "Price (relative)" parameter, reflecting a strategic emphasis on cost-effectiveness and financial feasibility. Therefore, the significance of each parameter and its respective weight is context-dependent, and the prioritization can vary based on the specific goals and priorities of the decision-makers involved in the evaluation process. Lastly, the "Maximum Working Temperature [degC]" parameter, with the lowest weight of 0.036, indicates that changes in this factor have a relatively lower impact on the decision-making process. It's important to recognize that the suggested method allows for flexibility in adjusting these weightings based on the specific circumstances of each stakeholder. Given the broader applicability of this approach, stakeholders have the flexibility to customize the weights to align with the unique priorities and objectives of their individual cases. This adaptability ensures that the decision-making process remains dynamic and responsive to the diverse considerations that may influence the selection of solar panels across different scenarios.

#### 6.2.5 Using PROMETHEE II, TOPSIS, EDAS and VIKOR methods to arrange the alternatives based on rankings

The normalized decision matrix, *R*_*ij*_ given by equation (29) and the weight vector *w*_*i*_ given by equation (30) were used in corresponding algorithms of MCDM methods discussed in previous section to obtain final ranking by respective MCDM methods. The results obtained from respective methods are discussed in the following sections.

## 7 Results

The rankings obtained of selected models by different MCDM methods based on weight vector presented equation (30) are summarized in Table 3.

**Table 3:** Ranks of modules obtained from different MCDM methods.

Rank	PROMETHEE II	EDAS	TOPSIS	VIKOR
1	M1	M7	M1	M3
2	M7	M10	M2	M6
3	M8	M9	M5	M2
4	M2	M4	M8	M5
5	M5	M6	M9	M9
6	M10	M8	M7	M1
7	M9	M3	M10	M4
8	M6	M1	M6	M7
9	M3	M5	M4	M8
10	M4	M2	M3	M10

**Fig. 7:**
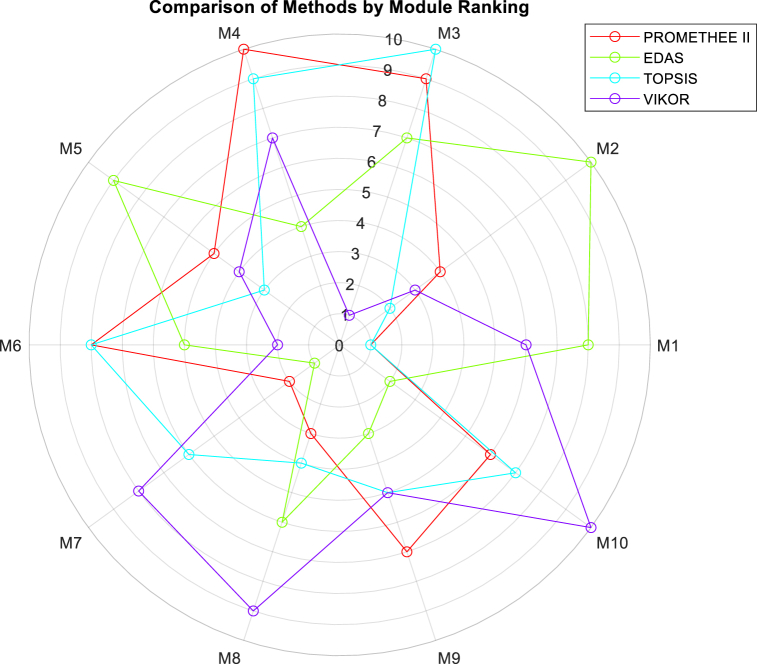
Radar chart for PVT collector selection based on weight vector of *w*_*i*_ = [0.166, 0.212, 0.190, 0.214, 0.036, 0.182].

It can be observed from Table 3 that the ranks of selected alternatives from various MCDM methodologies are relatively similar for TOPSIS and PROMETHEE II methods. A major difference between PROMETHEE-II and other methods considered in this paper is that PROMETHEE-II is an outranking method, whereas others are methods that rely on the Euclidean distance of alternatives. In EDAS, the ranks of alternatives are determined based on their distances from an average solution. TOPSIS and VIKOR, in contrast to EDAS, determine the ranks based on the Euclidean distance of alternatives from the ideal best and the ideal worst alternatives. VIKOR is an updated version of TOPSIS, which considers the ratio of the positive and negative ideal solution, thereby removing the drawback of the TOPSIS method. The variations in rankings among different modules (see Fig. 7) stem from the fact that each module excels in different aspects. For instance, M6's high electrical efficiency is certainly interesting for applications focused on electricity generation, but it may not perform as well in terms of thermal efficiency, which affects its overall ranking. Additionally, factors like maximum working temperature and price play a significant role in assessing the practicality and economic feasibility of implementing a specific PVT module. In essence, our approach aims to strike a balance between various criteria, acknowledging that different applications and scenarios may prioritize different aspects of PVT module performance. This comprehensive evaluation ensures that the selected module aligns optimally with the specific needs and objectives of the renewable energy system in question.

The price of PVT collectors plays a crucial role in their selection for a variety of reasons. Firstly, the initial investment cost is a significant factor in the decision-making process. Lower-priced PVT collectors can make solar energy more accessible to a wider range of consumers, thereby promoting the adoption of renewable energy. Secondly, the price is directly linked to the return on investment. The lower the cost of the PVT collectors, the quicker the payback period, making the investment more attractive. Therefore, when selecting PVT collectors, a balance must be struck between price, quality, and long-term benefits. For this reason, an additional example is considered here by increasing the weight of the relative price of the module in the weight vector presented in equation (30) from 0.182 to 0.410. The revised weight vector is presented in equation (31).

(31) *w*_*i*_ = [0.100, 0.150, 0.130, 0.120, 0.090, 0.410]

The rankings obtained for selected models by different MCDM methods, based on the revised weight vector focused on relative price presented in equation (31), are summarized in Table 4.

**Table 4:** Ranks of modules obtained from different MCDM methods based on the revised weight vector focused on relative price.

Rank	PROMETHEE II	EDAS	TOPSIS	VIKOR
1	M1	M7	M1	M7
2	M7	M9	M2	M1
3	M2	M10	M5	M2
4	M5	M4	M7	M3
5	M8	M6	M10	M4
6	M10	M8	M8	M5
7	M6	M3	M6	M9
8	M9	M1	M4	M10
9	M4	M5	M9	M6
10	M3	M2	M3	M8

**Fig. 8:**
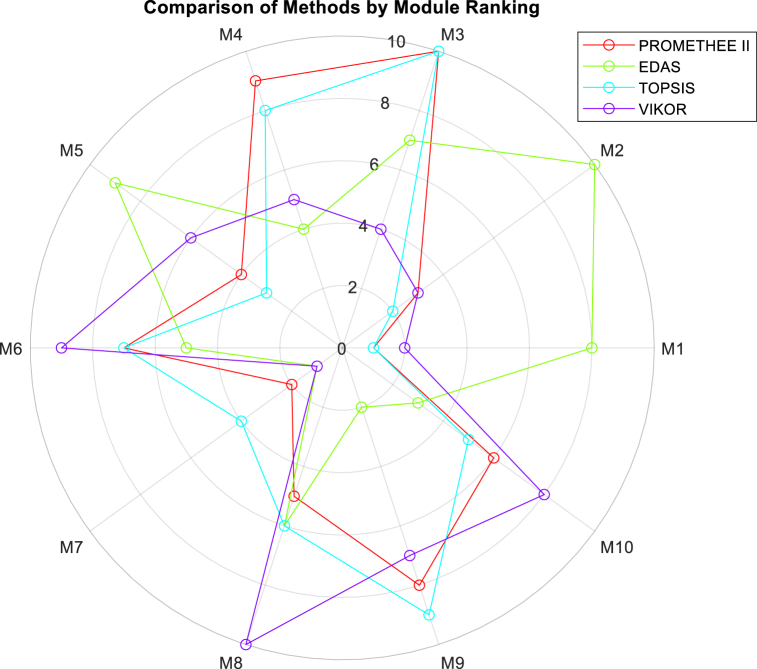
Radar chart for PVT collector selection based on weight vector of *w*_*i*_ = [0.100, 0.150, 0.130, 0.120, 0.090, 0.410].

Table 4 and Fig. 8 showcase the rankings of PVT modules obtained from various MCDM methods, all based on the revised weight vector that emphasizes the relative price. Each method, including PROMETHEE II, EDAS, TOPSIS, and VIKOR, offers its unique perspective on the modules' suitability concerning this weighted criterion. Notably, M1 consistently emerges as a top-ranking module across PROMETHEE II and TOPSIS methods, indicating its strong performance in terms of relative price compared to other modules. Meanwhile, M7 also maintains a prominent position across multiple methods, suggesting its favorable ranking in terms of price among the considered modules. Conversely, modules like M4 and M3 tend to have lower rankings across these methods, indicating comparatively weaker performance in relation to the emphasized criterion of relative price.

## 8 Conclusion

In this study, various MCDM techniques are proposed for the selection of PVT collectors to refine the selection of candidate PVT modules for hybrid solar systems. The Preference Ranking Organization Method (PROMETHEE)-II, Technique for Order of Preference by Similarity to Ideal Solution (TOPSIS) method, Evaluation Based on Distance from Average Solution (EDAS) method, and VIseKriterijumska Optimizacija I Kompromisno Resenje (VIKOR) MCDM techniques have been discussed. This study considers both qualitative and quantitative data sets within a MATLAB program to produce a PVT selection suitability index that allows for the simple selection of a PVT module. This study also considers the relative importance of each selected factor to be accounted for and considers this via both objective and subjective routes. MATLAB's inherent matrix operations make it particularly suited for multi-criteria decision-making methods, allowing for efficient computation and result visualization. For this study, MATLAB was leveraged due to its powerful analytical capabilities, ensuring a thorough and accurate assessment of PVT collectors based on selected criteria. The proposed method considers ten commercially available PVT collectors (modules) by avoiding conflict of interest and protecting the interests of manufacturers. The proposed methods are tested against a real-life situation in PVT module selection based on key performance indicators including electrical efficiency, collector thermal efficiency, maximum working temperature, and collector price per unit area. While the results from Euclidean distance-based methods like TOPSIS, EDAS, and VIKOR indicated M7 as the selected choice for the given scenario, one might question the reason behind its prominence despite not being the absolute best in any single category. Upon deeper inspection, M7 shows its true worth not just in individual parameters but in its holistic performance. It has a well-rounded balance between electrical efficiency, collector thermal efficiency, and operating temperatures. Moreover, the configuration and setup of M7 allow for efficient energy harvesting, even in conditions not represented by the standard test conditions. Its design emphasizes an optimal balance, ensuring its adaptability across varied operating scenarios. This versatility, coupled with its economic viability given its relative price, makes M7 a compelling choice for various applications in the renewable energy landscape. The methodologies developed in this study are poised to transform the selection process of PVT collectors for renewable energy systems, and the principles can extend beyond to areas such as PVT collector design.

## Data availability

Data will be made available on request.

## CRediT authorship contribution statement

**Sahand Hosouli:** Writing – review & editing, Supervision, Project administration, Methodology, Formal analysis, Conceptualization, Resources, Writing – original draft. **Nachiket Gaikwad:** Writing – original draft, Data curation, Formal analysis. **Shabahat Hasnain Qamar:** Investigation. **Joao Gomes:** Writing – review & editing, Resources, Funding acquisition, Conceptualization, Project administration.

## Declaration of competing interest

The authors declare that they have no known competing financial interests or personal relationships that could have appeared to influence the work reported in this paper.

## Abbreviations

**The following abbreviations are used in this manuscript**
PVT: Photovoltaic Thermal Collector
MCDM: Multi Criteria Decision Making
PROMETHEE-II: Preference Ranking Organization Method II
TOPSIS: Technique for Order of Preference by Similarity to Ideal Solution
EDAS: Evaluation Based on Distance from Average Solution method
VIKOR: VIseKriterijumska Optimizacija I Kompromisno Resenje
